# Active removal of anterior segment-migrated dexamethasone implant (Ozurdex^®^)

**DOI:** 10.3205/oc000195

**Published:** 2022-03-22

**Authors:** Jozef Adelson M. Depla, Marc Veckeneer, Isabel Bleyen

**Affiliations:** 1 Retina Service, AZ Monica Deurne Hospital, Deurne, Belgium; 2 Retina Service, ZNA Middelheim Hospital, Antwerp, Belgium; 3 Cornea Service, AZ Monica Deurne Hospital, Deurne, Belgium

**Keywords:** dexamethasone, implant, migration, Ozurdex

## Abstract

**Background:** Ozurdex^®^ (Allergan plc., Dublin, Ireland) is an intravitreal sustained-release dexamethasone (DEX) implant. The implant has been reported to migrate into the anterior chamber, potentially causing corneal decompensation. Prompt removal or relocation in the vitreous cavity is advised but troublesome due to its fragility. Several techniques exist, but elaborate setup and specialized surgical skills that are required may cause delay in treatment. We report a novel technique that avoids these shortcomings.

**Case presentation:** A 59-year-old woman presented to the emergency department with visual loss due to an anterior chamber-migrated DEX implant and corneal edema. Using an ophthalmic viscosurgical device (OVD) and a bent 19-gauge needle, the implant has promptly been removed in a one-minute procedure under topical anesthesia.

**Conclusion:** Aspirating an anterior chamber-migrated DEX implant using a 19-gauge bent needle is a cost-effective, time-efficient and safe technique, not requiring specialized surgical skills.

## Introduction

Since macular edema has been treated with an intravitreal sustained-release dexamethasone (DEX) implant (Ozurdex, Allergan plc., Dublin, Ireland), reports of implant migration into the anterior chamber have been published [1], [2], [3]. A recent retrospective review has estimated the risk of migration at 1.6%, with a higher rate of implant migration in vitrectomized eyes (4.8%) [4]. The risk factors for implant migration described in the literature are vitrectomy, presence of a patent iridotomy and previous cataract surgery with capsular or zonular defects or aphakia [1], [3], [5], [6]. Although implant migration is rare, it can have serious vision-threatening consequences. In a series of 12 patients, Gonçalves et al. reported that 91.7% of cases of implant migration developed corneal edema and more than one-third of them had this complication treated surgically [4]. In the largest series, Khurana et al. reported 15 patients [5]. Corneal edema developed in 14 of these patients, did not resolve in 10, and required subsequent corneal transplantation in 6 cases. Earlier removal reduced the likelihood of permanent corneal edema (0.5 days vs 5.5 days from diagnosis of migration to surgical removal of the implant; *P*=0.04). In their report, Khurana et al. oberserved corneal edema in patients with early migration in <3 weeks [5], which may be due to the chemical toxicity of the implant components (dexamethasone and lactic and glycolic acids) and the mechanical trauma caused by a rigid polyvinyl acid polymer. Removal of the implant is challenging and several procedures have been proposed, including removal using intraocular forceps [2], [4], [7]. As the DEX implant becomes friable over time, removal with forceps often fractures it into multiple small fragments. Passive expression of these fragments through the corneal incision by filling the anterior chamber with viscoelastic can be attempted, but is troublesome, especially in vitrectomized eyes. As there is no vitreous gel to act as a ‘cushion’ for the iris and lens, a high amount of viscoelastic is needed to obtain sufficient positive intracameral pressure for expression of the fragments. This manoeuvre deepens the anterior chamber and pushes the iris-lens diaphragma posteriorly. Apart from being painful for the patient under topical anesthesia, zonular support may be damaged. Another reported technique uses 20-gauge flexible cannulas (Abbocath; Becton-Dickinson, Franklin Lakes, NJ) connected to the extrusion system from the vitrectomy machine to aspirate the implant [4]. In addition to not being cost-effective and requiring referral of the patient to a specialized ophthalmology department, the use of a flexible cannula requires the DEX implant to be perfectly positioned with its smaller diameter towards the direction of the corneal incision. The manipulation needed to obtain this alignment can disintegrate the brittle implant, making removal more complicated. Herein we describe a simple surgical technique for DEX implant removal from the anterior chamber that obviates the need for general anesthesia and minimizes the required surgical equipment and skills.

## Case description

A 59-year-old woman presented to the emergency department with an anterior chamber-migrated DEX implant. She had a history of repeated intravitreal and subtenon’s triamcinolone injections prior to the first DEX implant for macular edema following complicated vitreoretinal surgery. The eye was pseudophakic with an intraocular lens (IOL) in the bag and intact posterior capsule. One month following the second intravitreal injection of Ozurdex, the patient underwent surgery for dislocation of the IOL. The lens and the capsular bag were removed via an anterior, corneal approach and a retropupilary iris-fixated IOL (Artisan^®^ aphakia model 205, Ophtec Inc., Groningen, The Netherlands) was implanted. Five days later, the Ozurdex implant migrated into the anterior chamber. The patient presented to the emergency department with visual loss due to corneal edema. Using topical anesthesia (oxybuprocaine 0.4%), a 2mm corneal incision was made. The anterior chamber was filled with a cohesive ophthalmic viscosurgical device (OVD) (ProVisc^®^, Alcon, Geneva, Switzerland) so that the smaller diameter of the implant was directed slightly towards the corneal incision. A 19-gauge bent needle was used to aspirate the implant. Following removal of the viscoelastic, sealing of the corneal incision was confirmed. At the conclusion of the surgery, 0.1 mg of cefuroxime (Aprokam^®^, Théa Pharma B.V., Haarlem, The Netherlands) was injected intracamerally. During the procedure, the patient did not report any discomfort or pain. The cornea cleared slowly over the next weeks, with visual acuity improving from counting fingers to 0.1.

### Detailed explanation of surgical technique

**Figure 1:**
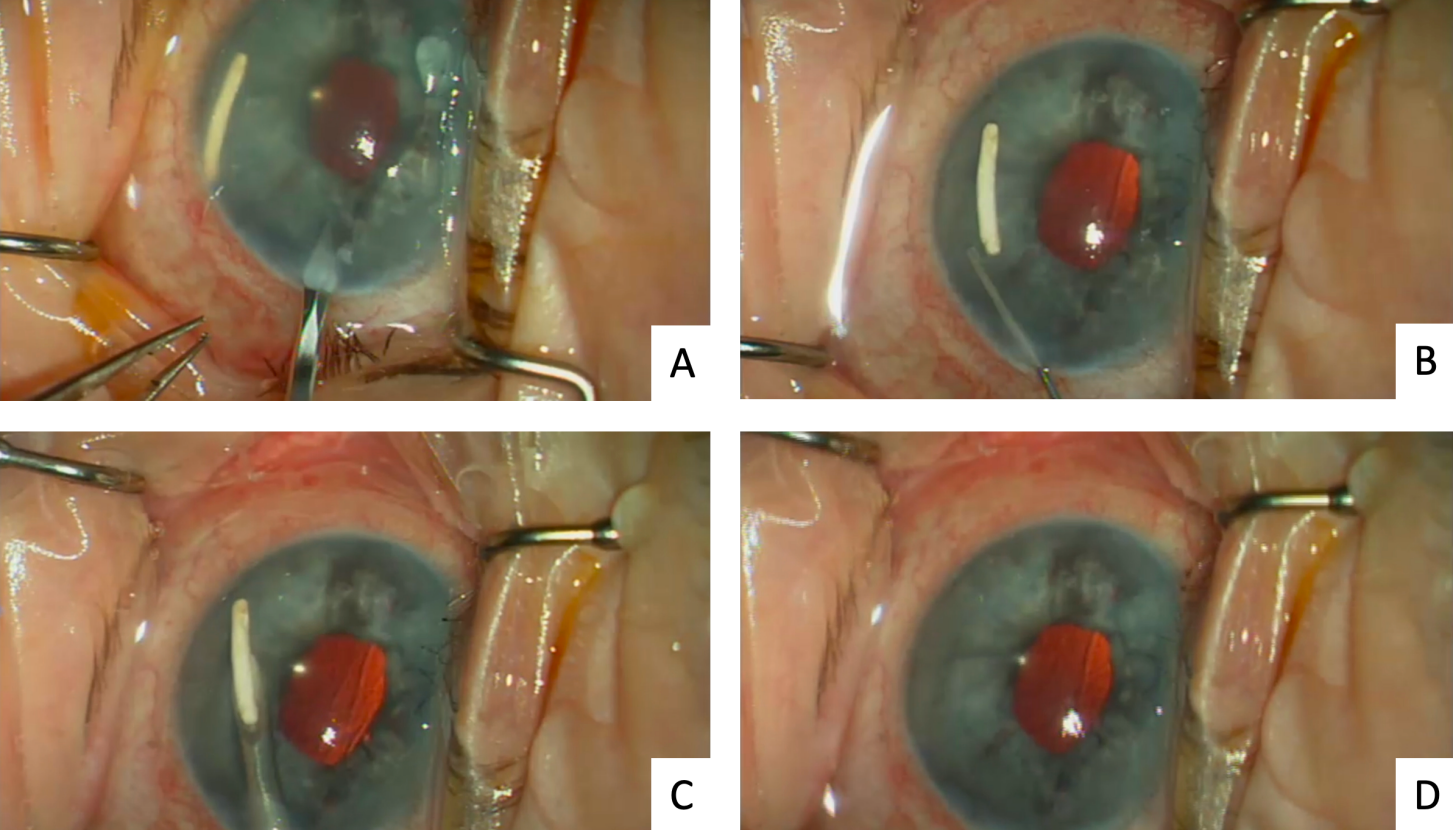
Surgical removal of the dexamethasone implant from the anterior chamber. A) Corneal incision. B) Positioning of the implant towards the incision using a cohesive OVD. C) Positioning of the 19-gauge needle around the implant. D) Final stage of the surgery.

Initially, a 2 mm clear corneal incision is made with a keratome blade (Figure 1 (Fig. 1), Attachment 1). The anterior chamber is filled with a cohesive OVD and the implant is oriented with the smaller diameter slightly towards the incision. A 19-gauge needle with luer lock is connected to a 3 ml syringe. Using a needle holder, the needle is bent approximately 30 degrees bevel up, as to provide easier manipulation for accessing the anterior chamber. After introducing the needle into the anterior chamber, it is positioned in the extension of the DEX implant. In this way, the implant causes an incomplete occlusion of the needle tip. Gentle aspiration is applied by pulling back the plunger of the syringe. Following this manoeuvre, the implant will be aspirated without touching it, so as not to cause fragmentation. To complete the case, the OVD is removed from the anterior chamber with a simcoe cannula. The 2 mm clear corneal incision generally does not need suturing.

## Discussion and conclusions

To give the patient the best odds for corneal recovery, reducing the delay between diagnosis and removal of the migrated implant is crucial. Complicated procedures requiring anesthesia and extensive surgical equipment should be avoided. Especially in vitrectomized eyes, passive expression leads to a painful deepening of the anterior chamber and is prone to damaging the zonular fibers.

Our procedure obviates the need for a vitrectomy machine and can be performed under topical anesthesia. The implant just needs to be slightly oriented towards the incision as the use of a rigid needle greatly increases manoeuverability. The skills needed for this procedure are within the scope of any surgeon performing intraocular surgery.

## Abbreviations

- DEX: dexamethasone
- IOL: intraocular lens
- OVD: ophthalmic viscosurgical device

## Notes

### Ethical approval

This study was approved by the institutional review board of AZ Monica Deurne Hospital (ethical committee approval number 455).

### Informed consent

Written informed consent was obtained from the patient for publication of this case report and any accompanying images.

### Authors’ contribution

JD has carried out the procedure and written the article. MV and IB have substantially revised it.

### Funding

No funding was received for this work.

### Competing interests

The authors declare that they have no competing interests.

## Supplementary Material

Video: Removal of anterior segment-migrated dexamethasone implant
